# Expression of the Body-Weight Signaling Players: GDF15, GFRAL and RET and their clinical relevance in Gastric Cancer

**DOI:** 10.7150/jca.55511

**Published:** 2021-06-04

**Authors:** Karolina Buchholz, Paulina Antosik, Dariusz Grzanka, Maciej Gagat, Marta Smolińska, Alina Grzanka, Arkadiusz Gzil, Anna Kasperska, Anna Klimaszewska-Wiśniewska

**Affiliations:** 1 Department of Clinical Pathomorphology, Faculty of Medicine, Collegium Medicum in Bydgoszcz, Nicolaus Copernicus University in Toruń, Poland.; 2 Department of Histology and Embryology, Faculty of Medicine, Collegium Medicum in Bydgoszcz, Nicolaus Copernicus University in Toruń, Poland.

**Keywords:** GDF15, GFRAL, RET, gastric cancer, prognostic factor

## Abstract

The existence, the functional role and clinical relevance of GDF15 and its signaling through a GFRAL/RET-dependent complex in gastric cancer (GC) and other human tumors remain to be elucidated, despite the widespread recognition of obesity as an important cancer-predisposing factor. Therefore, we aimed to analyze the expression levels of GDF15, GFRAL and RET in GC tissues in relation to each other and clinicopathological features, including patient survival, in order to establish a potential implication of the body-weight signaling pathway in the pathology and clinical outcome of GC. Protein expression was examined by immunohistochemistry on tissue microarrays containing 104 and 30 consecutive GC and normal gastric mucosa samples, whereas gene expression data for The Cancer Genome Atlas cohort of 413 GC patients were obtained from public sources. We found that the protein expression of GDF15, GFRAL and RET was significantly elevated and positively correlated in our set of GC tissues, which was reflected in their tendency to be overexpressed in low-grade and intermediate-grade tumors rather than high-grade ones. No other relationships between the expression status of the examined proteins and clinicopathological characteristics of GC patients were found. Through *in silico* data analysis, we showed that high *GDF15* expression was associated with better overall survival (OS) of GC patients, whereas the opposite was true for high levels of *GFRAL* or *RET*. Specifically, *GFRAL* and *RET* emerged as independent prognostic factors associated with poor OS. Furthermore, high combined expression of the three markers: *GDF15+GFRAL+RET* was significantly associated with reduced OS, and it was an independent prognostic factor of borderline significance in terms of OS, when adjusted for covariates. If validated in large-scale studies, the individual and combined expression of *GDF1*5, *GFRAL* and *RET* may provide significant clinical implications for the prognosis prediction of GC patients.

## Introduction

Gastric cancer (GC) is the fifth most common malignant neoplasm and simultaneously the third leading cause of cancer death worldwide 1. Although the GC incidence decreased in the last decades, the mortality is as much as 8.2% of all cancer deaths and the general five-year survival rate is approximately 28% 1, 2. The above data indicate that most of the diagnosed cases are in advanced stages. GC two-fold more frequently affects men than women 3. Moreover, the incidence and mortality rates are strongly associated with the geographical position and thus also with culture, diet, and economic development of the region. Most new cases and deaths are observed in countries of Eastern and Central Asia with the highest incidence and mortality rates in the Republic of Korea (39.6 per 100 000 cases) and Mongolia (25 per 100 000 cases), respectively. The lowest rates are noted in Northern America, Northern Europe and Africa 4, 5. According to Lauren's classification, there are three histological subtypes of gastric cancer: intestinal, diffuse and mixed 6, 7. The intestinal type consists of well-differentiated tumor cells with a tendency to form tubular or glandular structures. It is often associated with intestinal metaplasia triggered by chronic *Helicobacter pylori* infection. By contrast, the diffuse type is characterized by poorly differentiated tumor cells that lack adhesion and infiltrate individually or in small clusters throughout the gastric mucosa 8. The mixed type of gastric cancer is made up of both intestinal and diffuse types.

The growth differentiation factor 15 (GDF15), also known as macrophage inhibitory cytokine‑1 (MIC-1), is a protein encoded by the *GDF15* gene, which is located on chromosome 19p13.1-13.2 9. Originally, GDF15 has been described as a member of transforming growth factor β (TGF-β) superfamily 9-11. However, the results of recent studies have shown that GDF15 binds to the glial cell-derived neurotrophic factor (GDNF) family receptor α-like (GFRAL), not to TGF-β receptors as previously thought, which allowed to reclassify GDF15 as a member of the GDNF family of ligands (GFL) 12. Under physiological conditions, GDF15 is expressed in many tissues, including the placenta, prostate, kidney, colon, and liver 13. It has been shown that its level significantly increases in response to cellular stress or tissue injury. Consequently, the overexpression of GDF15 was found in cardiovascular disease 14, 15, chronic kidney disease 16, diabetes 17, inflammation 18, cancer-induced cachexia 19 and erythroid related disorders 20, but it was also observed after exhaustive physical exercises 21, 22. Moreover, GDF15 plays a key role in metabolism regulation as its high level is usually associated with body weight loss 12, 23. The GDF15 expression changes have also been detected in many tumors, including gastric cancer. The study conducted by Liu et al. has demonstrated that *GDF15* mRNA expression levels in gastric cancer tissues and GDF15 protein levels in peripheral blood of GC patients are significantly higher in comparison to healthy individuals 24. Additionally, some authors have reported that the high serum levels of GDF15 in GC patients are associated with tumor invasion and lymph node metastasis 25. These results indicate that GDF15 has a potential diagnostic and prognostic value for GC.

Mature GDF15 interacts with highly specific extracellular GFRAL receptor identified recently by four independent research groups 12, 26-28. The highest expression of GFRAL has been detected in neurons of brainstem area postrema and nucleus of the solitary tract in mice and humans 27, but its presence has also been confirmed in human adipose tissue 29. Recent studies have shown that GFRAL is the only member of the GNDF family receptor-α (GFRα) capable of binding GDF15 ligand. Moreover, to activate intracellular signaling by GDF15, the interaction between the GDF15-GFRAL complex and the rearranged during transfection (RET) tyrosine kinase co-receptor is needed 12, 27, 28.

RET is a transmembrane receptor encoded by *RET* protooncogene located on chromosome 10q11.2, and is mainly related to the development of the nervous system, the urogenital system and cells derived from the neural crest 30, 31. RET is composed of an extracellular cadherin-like domain binding GFL-GFRα complex, a transmembrane region and an intracellular tyrosine kinase domain 32. The ligand binding to RET results in receptor autophosphorylation and later activation of multiple downstream pathways, including Ras/mitogen-activated protein kinase (MAPK), phosphatidylinositol-3-kinase (PI3K)/Akt and phospholipase C-γ (PLC-γ) pathways promoting cell cycle progression, proliferation, migration, survival, and differentiation of cells 33. Because of the important role which RET plays in crucial cellular processes, chromosomal rearrangements, point mutations and overexpression of the *RET* gene can lead to the development and progression of many tumors, including gastric cancer 34. The carcinogenesis can also be promoted indirectly through GFL-GFRα-RET-dependent modulation of the tumor microenvironment what makes this signaling a potentially valuable molecular target for cancer therapy 35.

This study aimed to evaluate the immunohistochemical expression of GDF15, GFRAL and RET proteins both in the GC tumors and in the normal gastric tissues. The research also included the reference of obtained results to clinicopathological data and the analysis of the correlation between chosen proteins. Moreover, based on The Cancer Genome Atlas (TCGA) data, the expression of *GDF15*, *GFRAL* and *RET* genes was assessed in the context of patient survival.

## Materials and methods

### Material

The study was conducted on archival formalin-fixed, paraffin-embedded (FFPE) tissue samples derived between 2007 to 2015 from 104 patients diagnosed with GC at the Department of Clinical Pathomorphology, Collegium Medicum in Bydgoszcz, Nicolaus Copernicus University in Toruń. All tumors were reclassified in accordance with the standardized TNM 8^th^ edition classification of the American Joint Committee on Cancer (AJCC) criteria 36. Moreover, all gastric cancer tissues were classified as intestinal, diffuse or mixed type based on Lauren's system (Table 1). The study group involved both non-invasive (carcinomas *in situ*, n=8) and invasive (adenocarcinomas, n=96) gastric cancer samples, while the control group included 30 normal gastric mucosa tissues from the patients who underwent endoscopy between 2016 and 2017. Patients were excluded when they had a second primary carcinoma or received prior treatment. The same cohorts of patients and tissue samples have been included in our previously published report 37.

### Ethics statement

The study protocol was approved by the Bioethics Committee at Collegium Medicum in Bydgoszcz of Nicolaus Copernicus University in Toruń (no. 76/2018).

### Methods

#### Tissue microarrays preparation

Tissue microarrays (TMAs) were constructed as previously described 37. In brief, representative areas within the tissue samples, containing at least 80% of tumor cells, were identified under the microscope using archival tissue sections stained with hematoxylin and eosin (H&E). Then, two cylindrical 2 mm fragments of FFPE tissue specimens were removed from every donor block and inserted into recipient blocks with an automated tissue arrayer (TMA Master, 3DHISTECH Ltd., Hungary). The prepared TMA blocks were routinely sectioned at 4-µm thickness using a rotary microtome (Accu-Cut^®^ SRM^TM^200, Sakura Finetek, Torrance, CA, USA) and placed on high-adhesive glass slides (Superfrost Plus; MenzelGlasser, Germany) afterward.

#### Immunohistochemical staining

The TMA slides were subjected to immunohistochemical staining according to the previously described protocol 37. To evaluate GDF15, GFRAL and RET proteins expression, the primary antibodies including rabbit polyclonal anti-GDF15 (1/100, 45 min; cat. no: HPA011191, Sigma-Aldrich, Germany), rabbit polyclonal anti-GFRAL (1/200, 30 min; cat. no: HPA047372, Sigma-Aldrich, Germany) and rabbit monoclonal anti-RET (1/250, 30 min; cat. no: HPA008356, Sigma-Aldrich, Germany) were applied. The labeling conditions were standardized based on data available in The Human Protein Atlas (http://www.proteinatlas.org) and instructions provided by the manufacturer of antibodies. To detect antigen-antibody complexes, EnVision FLEX+ System (Dako, Agilent Technologies, Santa Clara, CA, USA) was used.

#### Evaluation of immunohistochemical reactions

The immunohistochemical expression of GDF15, GFRAL and RET proteins was evaluated by two independent pathologists using modified immunoreactive score of Remmele and Stegner (IRS) 38. The staining score (0-12) was determined by multiplying the percentage of positively stained cells/tissue area (0: 1-9%; 1: 10-20%; 2: 21-50%; 3: 51-80%; 4: 81-100%) and the expression intensity (0: negative, 1: weak, 2: moderate, 3: strong). The analysis was conducted at 20× original objective magnification in three randomly selected fields of view, and the final staining score was presented as a mean of three results. For GDF15 and GFRAL proteins, the staining score below 4 was considered as negative (low expression/without overexpression), whereas 4 or more as positive (overexpression). In the case of RET protein, the results below 9 were interpreted as negative, while 9 or more were defined as positive.

#### *In silico* analysis

The gene expression data for TCGA cohort of 413 gastric adenocarcinoma patients were downloaded through UCSC Xena Browser (http://xena.ucsc.edu/). The study included all TCGA adenocarcinoma samples for which expression data of selected genes were available. RNA-sequencing (RNA-seq) data for *GDF15*, *GFRAL* and *RET* were normalized via upper quartile normalization (UQ) and DESeq2 normalization (DESeq2; the data provided as Supplementary data), and finally dichotomized into high level expression and low level expression groups according to cutoff points determined with the cutp function of the *Evaluate Cutpoints* application 39. In order to determine the prognostic significance of *GDF15+GFRAL+RET* coexpression*,* the combined variables were assessed by adding their respective expression values, and their sum was dichotomized (UQ: < 18.02 or ≥ 18.02; DESeq2: < 18.11 or ≥ 18.11 using the *Evaluate Cutpoints* software 39) for purposes of statistical analysis. Twenty (4.84%) patients who died on the day of surgery were excluded from survival analysis. Two normalization methods were applied to exclude any bias originating from the data normalization.

#### Statistical analysis

Statistical analysis was carried out with GraphPad Prism (version 7.0; GraphPad Software, La Jolla, CA, USA) and *SPSS* software packages (version 26.0, IBM Corporation, Chicago, IL, USA). Data normality was assessed using the Shapiro Wilk test. Continuous variables were compared using the Mann-Whitney, whereas categorical variables with the chi-square test or Fisher's exact test, where appropriate. Correlations between the expression status of examined proteins were evaluated by the Spearman correlation coefficient. The survival data analysis was performed by the Kaplan-Meier method, and the differences between the survival curves were assessed using the Mantel-Cox log-rank test. To estimate the hazard ratio (HR) with 95% confidence intervals (CI), multivariate Cox proportional hazards models were built on all significant covariates measured by univariate Cox analysis, which satisfied the proportional hazards assumption. As a result, covariates were: pT status (T1 + T2* vs.* T3 + T4) and pN status (N0 *vs.* N1-N3). The P value < 0.05 was considered statistically significant.

## Results

### GDF15 expression in gastric cancer and normal gastric mucosa: association with clinicopathological parameters

**Figure 1:**
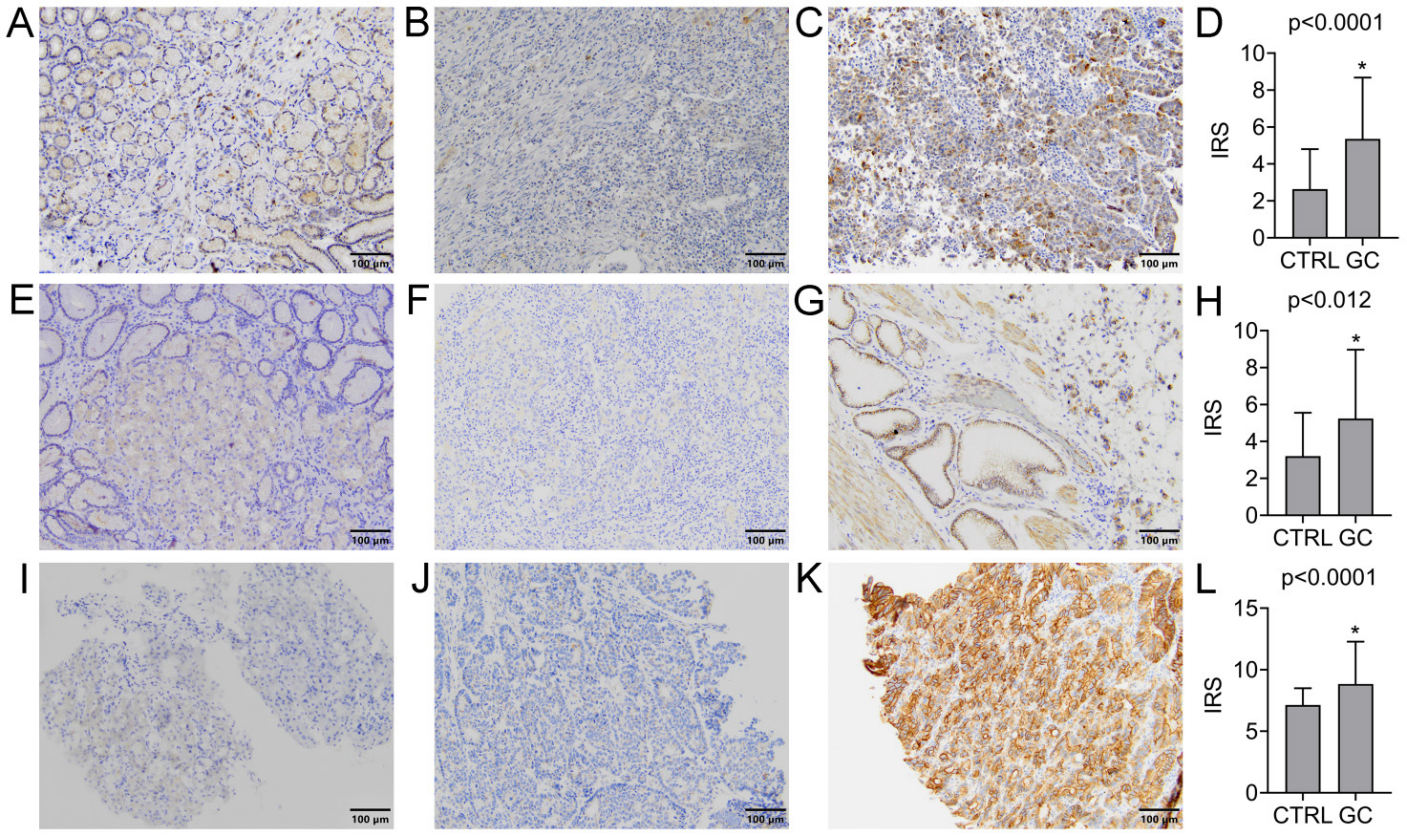
Immunohistochemical analysis of GDF15 (A-D), GFRAL (E-H) and RET (I-L) expression levels in normal and GC tissue samples. Immunohistochemical staining of the normal gastric mucosa (A, E, I) and GC tissue samples with low (B, F, J) and high (C, G, K) expression level of analyzed proteins. The increased expression level of GDF15 (D), GFRAL (H) and RET (L) in GC tissue compared to normal gastric mucosa samples (CTRL). Asterisk indicates statistical significance.

The expression of GDF15 was observed in the cytoplasm of gastric cancer cells and normal gastric mucosal cells. The positive rate was found in 72 (69.2%) GC cases, while the remaining 32 (30.8%) were negative. In turn, the positive expression was observed in 9 (30.0%) normal gastric samples, and negative in the other 21 (70.0%). Representative images of IHC staining for GDF15 are presented in Figure 1A-C. According to the above data, the expression level of GDF15 was significantly higher in GC tissues compared to the control group (*P < 0.0001*; Figure 1D). The high prevalence of GDF15 overexpression was noted in each histologic grade of GC that is well (100.00%), moderately (77.78%) and poorly (61.40%) differentiated tumors, and the observed differences were statistically significant (*P = 0.04*). According to Lauren's classification, GDF15 positivity was found in all histological types with high rates for mixed (77.78%) and intestinal (75.93%) types and a lower rate for diffuse type (58.54%), but without statistical significance (*P* = 0.16). Likewise, the association between overexpression of GDF15 and the location of the tumor in pylorus (80.95%) cardia (75.76%), fundus (63.16%) and antrum (50.00%) of the stomach was observed, however the differences did not reach statistical significance (*P* = 0.19). The expression status of GDF15 was not associated with other clinicopathological data, such as age, gender, pT status, pN status and tumor size (*P* > 0.05; Table 2).

### GFRAL expression in gastric cancer and normal gastric mucosa: association with clinicopathological parameters

GFRAL was expressed in the membrane of GC cells, as well as normal gastric mucosal cells. The positive and negative expression was found in 64 (61.54%) and 40 (38.46%) GC cases, while in the case of normal gastric tissues, it was 13 (43.33%) and 17 (56.67%) cases, respectively. Representative images of IHC staining for GFRAL are presented in Figure 1E-G. The difference in GFRAL expression level between GC tissues and control was statistically significant (*P = 0.012*; Figure 1H). The decrease in histological differentiation was strongly associated with lower rates of GFRAL-positive tumors. The GFRAL overexpression was more frequently detected in the well (100.00%) and moderately (80.00%) differentiated tumors than in the poorly differentiated ones (45.61%), and these differences were highly statistically significant (*P* = 0.0002). Moreover, the statistically significant difference (*P = 0.01*) in the prevalence of positive GFRAL expression was noted in the mixed (77.78%) and intestinal (72.22%) types in comparison to the diffuse type (43.90%). In contrast, the immunoexpression of GFRAL did not differ significantly depending on the age, gender, pT status, pN status, location and tumor size (*P > 0.05*; Table 2).

### RET expression in gastric cancer and normal gastric mucosa: association with clinicopathological parameters

The RET immunoexpression was evaluated in the membrane and cytoplasm of GC cells and normal gastric cells. The overexpression of RET protein was confirmed for 47 (45.19%) GC tumors, while the other 57 (54.81%) presented negative expression. Representative images of IHC staining for RET are presented in Figure 1I-K. The expression level of RET was significantly higher in GC tissues compared to the control group (*P* < 0.0001; Figure 1L). According to grading criteria, the RET positivity was more often observed in the well (50.00%) and moderately (53.33%) differentiated tumors than in the poorly differentiated ones (38.60%), however without statistical significance (*P* = 0.16). The RET expression was not significantly associated with any remaining clinicopathological features (Table 2).

### Correlation between the expression of GDF15, GFRAL and RET in gastric cancer

**Figure 2:**
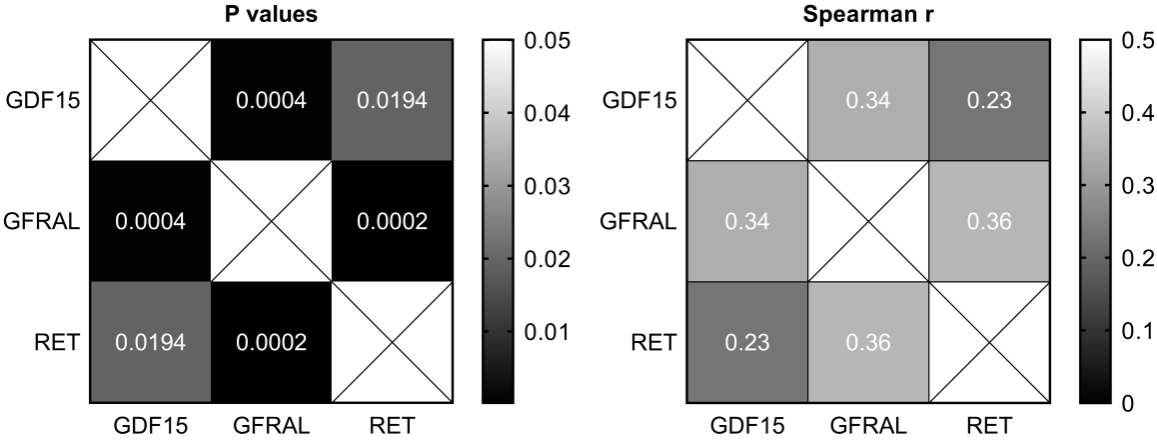
Heatmaps of Spearman's correlation between the expression of GDF15, GFRAL and RET in gastric cancer tissues.

There were moderate positive and statistically significant correlations between the expression of GDF15 and GFRAL (*P = 0.0004*, r = 0.34), as well as GFRAL and RET (*P = 0.0002*, r = 0.36), and a weak positive and statistically significant correlation between the expression of GDF15 and RET (*P = 0.02*, r = 0.23; Figure 2).

### *In silico* survival analysis

**Figure 3:**
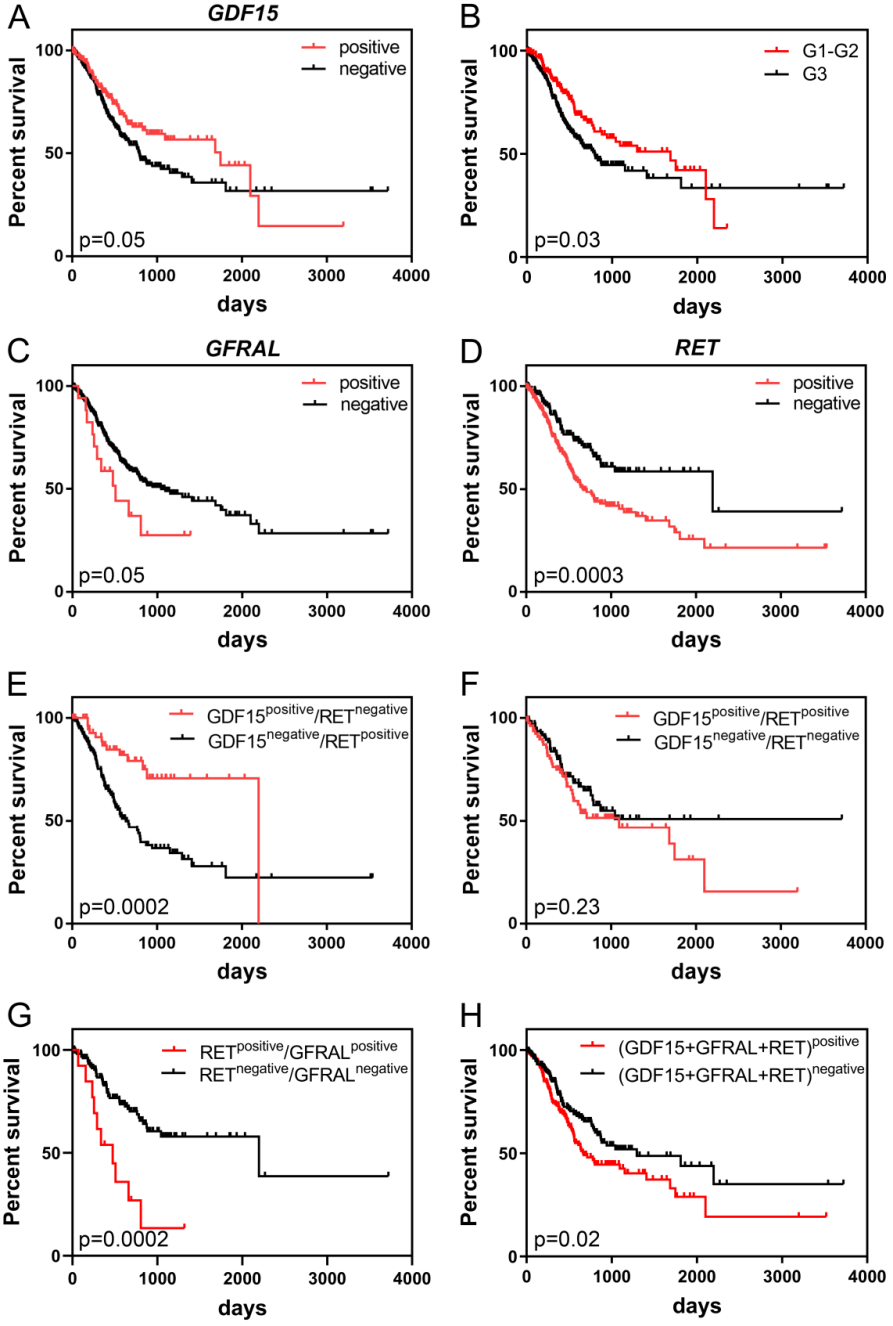
Kaplan-Meier curves displaying the overall survival of GC patients depending on *GDF15* expression (A), histologic grade of GC (B), *GFRAL* expression (C), *RET* expression (D), the combination of *GDF15* and *RET* expression (E, F), the combination of *RET* and *GFRAL* expression (G) and the sum of *GDF15, GFRAL* and *RET* expression (H) prepared based on the UQ-normalized RNA-seq data.

Kaplan-Meier survival analysis demonstrated that high *GDF15* expression was marginally significantly associated with better overall survival (OS) of GC patients (*P* = 0.05). The median survival times were 792 and 1747 days for low and high expression groups, respectively. It is worth noting, however, that patients with GDF15-overexpressing tumors had higher survival rates than GDF15-underexpressing tumors until day 2100, and after such period of time, this association was reversed (Figure 3A). Comparison of survival distributions between histologic grades yielded a similar pattern (*P* = 0.03; Figure 3B). In the univariate Cox analysis, overexpression of* GDF15* showed a borderline significant correlation with a better prognosis of GC patients (HR = 0.71, 95% CI 0.51-1.00, *P* = 0.05; Table 3). In turn, high *GFRAL* expression was marginally significantly associated with shorter survival time (507 days) of GC patients in comparison to those with its low expression level (1043 days, *P* = 0.05; Figure 3C), with HR calculation indicating an increase in relative risk of death from any cause of 1.81 (95% CI 0.98-3.35, *P* = 0.06). GFRAL overexpression appeared as an independent prognostic factor for poor OS in the multivariate Cox analysis (adjusted HR = 1.93, 95% CI 1.04-3.56, *P* = 0.04; Table 4). Furthermore, the TCGA dataset showed that *RET* overexpression was associated with a significantly shorter OS (2197 days vs. 669 days, *P* = 0.0003; Figure 3D; HR = 1.87, 95% CI 1.33-2.64, *P* = 0.0004; Table 3). Importantly, in multivariate Cox analysis, high *RET* expression persisted as a negative prognostic factor for OS, after adjustment for pN, and pT (HR = 1.80, 95% CI 1.26-2.56, *P* = 0.001; Table 4).

When we considered *RET* and *GDF15* together by Kaplan-Meier analysis, cases with both high *GDF15* expression and low *RET* expression had markedly longer OS than those with simultaneous low *GDF15* expression and high *RET* expression (2197 days vs. 633 days, *P* = 0.0002; Figure 3E). Furthermore, patients whose GC expressed both *RET* and *GDF15* at a high level had a shorter median OS compared to those whose GC expressed both *RET* and *GDF15* at a low level (1095 days *vs.* not reached, *P* = 0.23; Figure 3F)*,* and the survival benefit of *GDF15* overexpression visibly, but not significantly (*P* = 0.18) decreased when *GDF15* high expression was accompanied by *RET* overexpression (from 1747 days to 1095 days)*.* Moreover, patients whose GC simultaneously expressed *RET* and* GFRAL* at a high level had significantly shorter survival time compared to those patients whose tumor tissue expressed both these markers at a low level (474 days *vs.* 2197 days, *P = 0.0002*; Figure 3G). Finally, high combined expression of the three markers: *GDF15+GFRAL+RET* was significantly associated with reduced OS (669 days *vs.* 1294 days, *P* = 0.02; Figure 3H; HR = 1.45, 95% CI 1.06-1.99, *P* = 0.02; Table 3), and it was an independent prognostic factor of borderline significance in terms of OS, when adjusted for covariates, including pN and pT (HR = 1.35, 95% CI 0.98-1.86, *P = 0.07*; Table 4).

All of the above presented survival analyses are based on the UQ-normalized RNA-seq data. In the case of *GDF15* and *RET*, the results were quite similar for DESeq2-normalized data (Data S1; Figure S1A, D, E, F and H; Table S1 and S2). In the case of *GFRAL*, the results were somewhat less similar for DESeq2-normalized data, however still high levels of *GFRAL* were associated with worse OS, but with less statistical significance (Data S1; Figure S1B, C and G). These differences in analysis were most probably due to that in the TCGA cohort (n = 413), there were a lot of cases with zero read counts for *GFRAL* gene (n = 355).

## Discussion

GC is one of the most common cancers in the world and due to the lack of effective biomarkers for early detection, it is usually diagnosed at advanced stages. Therefore, it is so essential to broaden our knowledge about factors involved in GC pathogenesis and progression, which could improve the diagnosis process and simultaneously help in the development of new therapeutic strategies.

In recent years, numerous studies have been carried out to evaluate the expression level and the role of GDF15 in the biology of many types of human cancers, including gastric cancer. Although there is no clear consensus about the GDF15 expression level in gastric tumors compared to normal gastric tissues, most scientific reports have indicated a higher level of this protein in tumors than in normal tissues 40, 41, which is also consistent with our results. Whereas, in the case of *GDF15* mRNA in gastric tumors and protein levels in peripheral blood of GC patients, the data converge and present increased levels of GDF15 in patients with GC in comparison to the healthy population 24. Several attempts have also been made to investigate the correlation between GDF15 expression in GC tumors and clinicopathological features of patients 42-44. The scientists agree that the overexpression of GDF15 is strongly associated with the degree of tumor differentiation, which is also confirmed by our results. In our cohort, the GDF15 positivity rate more frequently occurred in the well differentiated cancer cells (100.00%) than in the moderately (77.78%) and the poorly differentiated ones (61.40%). Likewise, the analysis of the TCGA dataset also confirmed that *GDF15* overexpression was most common in G1-G2 gastric tumors (Table S3 and S4). A similar relationship has been observed by Park et al. 43 and Yang et al. 44, while Baek et al. presented inverse correlation 42. According to the report by Park 43, the positive GDF15 expression was also associated with T and N status, nevertheless, our own and TCGA data (Table S3 and S4) did not confirm this observation. Furthermore, it has been reported that the intestinal histological type of GC shows markedly higher GDF15 expression level compared to the diffuse histological type 43. A similar trend was also observed in our cohort, but the data were only marginally significant (intestinal type: 75.93%, diffuse type: 58.54%, *P = 0.08*). A partial variance of presented data may result from differences in methodology, including IHC scoring systems or specificity of antibodies used in the experiments, but also from variables such as ethnicity-related differences in gastric cancer biology, case numbers, distribution of the clinicopathological data or type of applied control tissue (normal gastric tissue harvested from healthy individuals vs. normal tissue obtained from areas adjacent to gastric tumors).

The function of GDF15 in the pathogenesis and progression of gastric cancer remains unclear since the experimental data support both the antitumorigenic or pro-tumorigenic activity of this multifunctional cytokine. The recent finding has demonstrated that GDF15 overexpression could be induced by tumor suppressor CXXC4 as a pro-apoptotic response in mutated gastric cells 45. In turn, GDF15 was found to be an important factor in cancer-stromal interaction in diffuse GC, as it contributed to NIH3T3 fibroblast activation by stimulating proliferation and up-regulating gene expression of extracellular matrix proteins 25. Previous studies have also indicated that GDF15 is involved in invasive and metastatic progression of GC via up-regulating the urokinase-type plasminogen activator system 46 and transactivation of ErbB2 47. The role of GDF15 in inducing cancer stem like cells in GC has been reported in the *in vitro* study of Guo et al., who showed that GDF15 overexpression promoted CSC-like properties of MGC803 cells, such as spheroid and soft-agar colony forming abilities 48. Besides, it has been reported that expression levels of GDF15 decreased significantly in GC patients who had a significant improvement after chemotherapy but remained high in pre-treatment patients and those with no significant improvement. This suggests that GDF15 overexpression in GC tissues resulted in a high level of circulating protein, what makes it potentially attractive biomarker for diagnosis, evaluation of treatment efficacy, prediction of GC progression, and monitoring of disease recurrence 41. In addition, a link between GDF15 expression status and GC prognosis with respect to patient's survival has been demonstrated. Blanco-Calvo et al. have found that increased serum levels of GDF15 protein were associated with shorter progression-free survival and overall survival of GC patients 49. In contrast, our Kaplan-Meier survival analysis of TCGA data showed that patients whose gastric cancer expressed a high level of *GDF15* mRNA had markedly longer median overall survival than those patients whose tumors expressed *GDF15* at a low level (1747 days *vs.* 792 days, *P = 0.05*; HR = 0.71, 95% CI 0.51-1.00, *P = 0.05*). This suggests the positive prognostic impact of a high tissue level of *GDF15* for the survival of GC patients. Of note, however, patients with GDF15-overexpressing tumors had higher survival rates than GDF15-underexpressing tumors until day 2100, and after such period of time, this association was reversed. It has previously been suggested that GDF15 may act as a tumor suppressor at the early stages of tumor development and progression, and later becoming a tumor promoter as the tumor progresses into a malignant phenotype 50. However, our remaining results did not support this thesis. Due to the lack of survival data of our cohort, which is an obvious limitation of the present study, we failed to validate a prognostic impact of* GDF15* at the tissue protein level, therefore further studies on GC clinical samples are necessary.

GFRAL is a distant member of the TGF-β family of receptors, which has been recently identified as a new regulator of body weight and as the bona fide receptor mediating the metabolic effects of GDF15. However, it remains to be established whether the impact of induced GDF15 overexpression in pathological conditions such as cancer still depends on GFRAL 51. To our best knowledge, the current study is the first which evaluates GFRAL expression in gastric cancer tissue samples. According to our data, the GFRAL expression was found to be significantly higher in gastric tumors compared with normal gastric mucosa, pointing out the role of this protein in the GC pathology. Furthermore, the correlation analysis between the expression status of GFRAL and clinicopathological characteristics of GC patients showed a markedly lower GFRAL expression level in poorly differentiated gastric cancer cells compared to those with more differentiated histology. In the present study, the relationship between GFRAL expression and Lauren's classification was also statistically significant, whereby the frequency of its overexpression was lower in diffuse gastric cancer type (43.90%) than in intestinal (72.22%) and mixed types (77.78%). In our cohort, a very similar rate of GFRAL positivity in the intestinal-type and mixed-type gastric carcinomas is probably related to the fact that our mixed-type cases are characterized by a predominance of intestinal histological component (mixed-predominantly intestinal type). Furthermore, based on the bioinformatic data, we found that the high *GFRAL* expression group showed shorter median OS than the low expression group (507 days *vs.* 1043 days, *P* = 0.05; HR = 1.81, 95% CI 0.98-3.35, *P =* 0.06). Importantly, *GFRAL* overexpression appeared as an independent prognostic factor for poor OS in the multivariate Cox analysis (adjusted HR = 1.93, 95% CI 1.04-3.56, *P* = 0.04). Whether GFRAL expression may be indeed an unfavorable prognostic factor for GC awaits stronger evidence both at mRNA and protein levels. Future evaluation of the role of GFRAL and associated signaling pathways in the biology of GDF15-overexpressing GC cells is also eagerly anticipated.

Recent findings have shown that GDF15-induced body-weight signaling pathway requires the interaction of GFRAL with the coreceptor RET 27. RET has been implicated in the activation of multiple signaling pathways promoting cell cycle progression, cell proliferation, migration, survival, and differentiation, and therefore, in the pathogenesis and metastatic progression of various cancers 35. However, apart from reports showing RET as a well characterized contributor to the neoplastic transformation, acting as an oncogenic driver, there are also those presenting this protein as a tumor suppressor 52. Here, we found that RET was significantly upregulated in GC tissues in relation to normal gastric mucosa, supporting the similar findings of Zhang et al., and their conclusion that RET overexpression may be one of the molecular changes driving gastric carcinogenesis 34. This is additionally underpinned by the fact that the new diagnostic method developed by Zhang et al., based on contrast-enhanced computed tomography (CECT) combined with liposome-encapsulated targeted contrast agents containing tyrosine kinase inhibitors of GC, including RET, allowed for the effective diagnosis of patients in the early stage of gastric cancer 53. To our knowledge, the cited study of Zhang et al. is the only one existing in the scientific literature which assessed by IHC the expression level of RET protein in the series of GC tissues. In turn, our study is the first, which correlated this expression with the clinicopathological parameters of GC patients, nevertheless, no significant relationships were observed. However, based on TCGA dataset, we found that high *RET* expression was significantly associated with reduced OS (HR = 1.87, 95% CI 1.33-2.64, *P* = 0.0004). The median survival times were 669 days and 2197 days for high and low expression groups, respectively. When examined in the multivariate Cox analysis, high *RET* expression persisted as a negative prognostic factor for OS of GC patients (adjusted HR = 1.80, 95% CI 1.26-2.56, *P =* 0.001). Further studies are warranted to validate its prognostic value for GC patients at the protein level.

The results of the present research and those of other authors have shown that GDF15 and RET proteins are individually overexpressed in GC tissues. However, as mentioned above, neither GRFAL expression nor especially the joint expression of GDF15, GFRAL and RET have been evaluated in gastric tumors, in the context of the potential implication of the body-weight signaling pathway in GC pathogenesis. As GDF15-GFRAL-RET axis plays a crucial role in the maintenance of energy metabolism through body weight regulation, it has been recently linked to carcinogenesis. Indeed, obesity is an important predisposing factor for cancer, including digestive system malignancies 54, and GDF15 is an etiological agent in cancer-related anorexia/cachexia syndrome (CACS) 55, as up-regulated GDF15 serum level was found to be a factor reducing appetite and body weight 19, and inhibition of GDF15-GFRAL activity prevents cancer cachexia 56. Additionally, it has been suggested that GDF15 probably directly modulates tumor biology and its microenvironment 51, 55. Whether this effect is also mediated via a GFRAL/RET-dependent complex remains to be clarified. It is expected that uncovering molecular details on GDF15 downstream signaling pathways in GC could potentially explain some of its pro and anti-tumorigenic roles 50. In order to investigate the potential relationship of GDF15, GFRAL, RET expression in GC, the strength and significance of their correlations were evaluated. We found that the expression of the examined proteins was positively and significantly correlated, which was also reflected in their tendency to be overexpressed in low-grade and intermediate-grade GC tumors rather than high-grade ones. Importantly, our bioinformatic analysis suggests that *GDF15* overexpression may have a positive impact on the survival of GC patients, but when *GDF15* overexpression is accompanied by the upregulation of its effectors from GDF15-GFRAL-RET axis, this seems to correlate with poorer OS. Indeed, patients whose GC expressed both *RET* and *GDF15* at a high level had a shorter median OS compared to those whose GC expressed both *RET* and *GDF15* at a low level (1095 days *vs.* not reached, *P = 0.23*)*,* and the survival benefit of *GDF15* overexpression visibly, but not significantly (*P = 0.18*) decreased when *GDF15* high expression was accompanied by *RET* overexpression (from 1747 days to 1095 days)*.* Furthermore, high combined expression of the three markers: *GDF15+GFRAL+RET* was significantly associated with reduced OS (669 days *vs.* 1294 days, *P* = 0.02; HR = 1.45, 95% CI 1.06-1.99, *P* = 0.02), and it was an independent prognostic factor of borderline significance in terms of OS, when adjusted for covariates (HR = 1.35, 95% CI 0.98-1.86, *P* = 0.07). One possible explanation for a worse survival of *GDF1*5+*GFRAL*+*RET*-coexpressing GC patients could be cancer-associated cachexia. However, due to the lack of available information regarding nutritional status, including the body mass index (BMI) and weight loss, both in our cohort and TCGA cohort, we were unable to verify this possibility, and further studies are required to address this issue.

The knowledge about the role of GDF15 and its signaling through a GFRAL/RET-dependent complex in gastric cancer is only beginning to be recognized, similar as in other human cancers. In the light of the current hypothesis that, due to very limited distribution of GFRAL, the effects of GDF15 on cancer cells are likely to be mediated through GFRAL-independent pathways 51, our study provides an interesting finding that the protein expression of GDF15, GFRAL and RET is significantly and jointly elevated in GC tissues. However, to validate their joint participation in GC, and to determine whether it is related to the metabolic and/or non-metabolic effects of GDF15, further investigations are required. Nevertheless, based on the present study, we conclude that the individual and combined expression of *GDF1*5, *GFRAL*, *RET* may provide significant clinical implications for the prognosis prediction of GC patients.

## Supplementary Material

Supplementary figures and tables.

## Figures and Tables

**Table 1:** Clinicopathological data of 104 patients with gastric cancer

Clinicopathological feature	No. of cases, *n* = 104	Percentage (%)
**Age (years)**		
≤ 60	27	26.0
> 60	77	74.0
**Gender**		
Male	73	70.2
Female	31	29.8
**Lauren's classification**		
Intestinal	54	51.9
Diffuse	41	39.4
Mixed	9	8.7
**Grading**		
G1	2	1.9
G2	45	43.3
G3	57	54.8
**pT status**		
Tis	8	7.7
T1	4	3.8
T2	27	26.0
T3	50	48.1
T4	15	14.4
**pN status**		
N0	40	38.5
N1	33	31.7
N2	27	26.0
N3	4	3.8
**Location**		
Cardia	33	31.7
Fundus	38	36.5
Antrum	12	11.5
Pylorus	21	20.2
**Tumor size (cm)**		
<5	41	39.4
≥5	63	60.6

**Table 2:** The immunohistochemical expression of GDF15, GFRAL and RET proteins and their relationship with clinicopathological parameters of GC patients

Clinicopathological feature	n (%), n = 104	GDF15 expression		P value	GFRAL expression		P value	RET expression		P value
		negative, n = 32	positive, n = 72		negative, n = 40	positive, n = 64		negative, n = 57	positive, n = 47	
										
**Age (years)**										
≤ 60	27 (25.96)	8 (29.63)	19 (70.37)	>0.99	11 (40.74)	16 (59.26)	0.82	15 (55.56)	12 (44.44)	>0.99
> 60	77 (74.04)	24 (31.17)	53 (68.73)		29 (37.66)	48 (62.34)		42 (54.55)	35 (45.45)	
**Gender**										
Male	73 (70.19)	20 (27.40)	53 (72.60)	0.26	28 (38.36)	45 (61.64)	>0.99	39 (53.42)	34 (46.58)	0.83
Female	31 (29.81)	12 (38.71)	19 (61.29)		12 (38.71)	19 (61.29)		18 (58.06)	13 (41.94)	
**Lauren's classification**										
Intestinal	54 (51.92)	13 (24.07)	41 (75.93)	0.16	15 (27.78)	39 (72.22)	0.01*	30 (55.56)	24 (44.44)	0.38
Diffuse	41 (39.42)	17 (41.46)	24 (58.54)		23 (56.10)	18 (43.90)		24 (58.54)	17 (41.46)	
Mixed	9 (8.65)	2 (22.22)	7 (77.78)		2 (22.22)	7 (77.78)		3 (33.33)	6 (66.67)	
**Grading**										
G1	2 (1.92)	0 (0.00)	2 (100.00)	0.04*	0 (0.00)	2 (100.00)	0.0002***	1 (50.00)	1 (50.00)	0.16
G2	45 (43.27)	10 (22.22)	35 (77.78)		9 (20.00)	36 (80.00)		21 (46.67)	24 (53.33)	
G3	57 (54.81)	22 (38.60)	35 (61.40)		31 (54.39)	26 (45.61)		35 (61.40)	22 (38.60)	
**pT status**										
Tis	8 (7.69)	2 (25.00)	6 (75.00)	0.92	4 (50.00)	4 (50.00)	0.25	4 (50.00)	4 (50.00)	0.89
T1-T2	31 (29.81)	10 (32.26)	21 (67.74)		15 (48.39)	16 (51.61)		18 (58.06)	13 (41.94)	
T3-T4	65 (62.50)	20 (30.77)	45 (69.23)		21 (32.31)	44 (67.69)		35 (53.85)	30 (46.15)	
**pN status**										
N0	40 (38.46)	12 (30.00)	28 (70.00)	>0.99	17 (42.50)	23 (57.50)	0.54	22 (55.00)	18 (45.00)	>0.99
N1-N3	64 (61.54)	20 (31.25)	44 (68.75)		23 (35.94)	41 (64.06)		35 (54.69)	29 (45.31)	
**Location**										
Cardia	33 (31.73)	8 (24.24)	25 (75.76)	0.19	13 (39.39)	20 (60.61)	0.96	20 (60.61)	13 (39.39)	0.70
Fundus	38 (36.54)	14 (36.84)	24 (63.16)		15 (39.47)	23 (60.53)		20 (52.63)	18 (47.37)	
Antrum	12 (11.54)	6 (50.00)	6 (50.00)		5 (41.67)	7 (58.33)		5 (41.67)	7 (58.33)	
Pylorus	21 (20.19)	4 (19.05)	17 (80.95)		7 (33.33)	14 (66.67)		12 (57.14)	9 (42.86)	
**Tumor size (cm)**										
<5	41 (39.42)	14 (34.15)	27 (65.85)	0.66	19 (46.34)	22 (53.66)	0.22	22 (53.66)	19 (46.34)	>0.99
≥5	63 (60.58)	18 (28.57)	45 (71.43)		21 (33.33)	42 (66.67)		35 (55.56)	28 (44.44)	

P value with statistical significance is marked in bold (chi-square test).

**Table 3:** Univariate Cox proportional hazards analysis for OS of TCGA patients with GC

Variable	Univariate analysis			
	HR	95% CI		*P*
		lower	upper	
*GDF15*	0.71	0.51	1.00	0.05
*GFRAL*	1.81	0.98	3.35	0.06
*RET*	1.87	1.33	2.64	**0.0004**
*GDF15+GFRAL+RET*	1.45	1.06	1.99	**0.02**
grading	1.44	1.03	2.02	**0.03**
pN status	2.09	1.39	3.14	**0.0004**
pT status	1.83	1.17	2.86	**0.01**
pM status	2.28	1.31	3.96	**0.003**

CI: confidence interval; GC: gastric cancer; HR: hazard ratio; OS: overall survival; TCGA: the Cancer Genome Atlas. Significant *p*-values (*P* < 0.05) are indicated in bold.

**Table 4:** Multivariate Cox proportional hazards models for OS of TCGA patients with GC

Variable	Multivariate analysis: *GFRAL*				Multivariate analysis: *RET*				Multivariate analysis: *GDF15+GFRAL+RET*			
	HR	95% CI		*P*	HR	95% CI		*P*	HR	95% CI		*P*
		lower	upper			lower	upper			lower	upper	
*GFRAL*	1.93	1.04	3.56	**0.04**	-	-	-	-	-	-	-	-
*RET*	-	-	-	-	1.80	1.26	2.56	**0.001**	-	-	-	-
*GDF15+GFRAL+RET*	-	-	-	-	-	-	-	-	1.35	0.98	1.86	0.07
pN status	1.85	1.21	2.85	**0.01**	1.80	1.18	2.76	**0.01**	1.79	1.17	2.74	**0.01**
pT status	1.46	0.91	2.36	0.12	1.43	0.89	2.30	0.14	1.47	0.92	2.37	0.11

CI: confidence interval; GC: gastric cancer; HR: hazard ratio; OS: overall survival; TCGA: the Cancer Genome Atlas. *p*-values adjusted for pN, pT, and each marker separately or the sum of respective expression values of each marker (according to column captions); the sum was dichotomized < 18.11 or ≥ 18.11 using the *Evaluate Cutpoints* software. '-' indicates variable was not included in multivariate analysis. Significant *p*-values (*P* < 0.05) are indicated in bold.
