# Supplementary material for: Expression of the Body-Weight Signaling Players: GDF15, GFRAL and RET and their clinical relevance in Gastric Cancer
Source: J Cancer. 2021 Jun 4;12(15):4698–709. doi: 10.7150/jca.55511 (PMC8210553; doi:10.7150/jca.55511)
Supplement: Supplementary file 1 — Supplementary figures and tables. [file jcav12p4698s1.pdf]

In silico survival analysis (DESeq normalization)

Kaplan-Meier survival analysis demonstrated that high *GDF15* expression was marginally significantly associated with better overall survival (OS) of GC patients. The median survival times were 794 and 1686 days for low and high expression groups, respectively (*P = 0.057*; Figure S1A). In the univariate Cox analysis, overexpression of *GDF15* was marginally significantly correlated with better prognosis of GC patients (HR = 0.72, 95% CI 0.51-1.01, *P = 0.06*; Table S1). Furthermore, high *GFRAL* expression was associated with shorter survival time (801 days) of GC patients in comparison to those with its low expression level (1043 days). However, this was not a significant survival difference (*P = 0.296*; Figure S1B). Likewise, in the univariate Cox proportional hazards model, *GFRAL* expression was not significantly associated with OS of GC patients (HR = 1,29, 95% CI, 0.80-2.06, *P = 0.30*; Table S1), and the multivariate Cox analysis demonstrated that *GFRAL* expression did not constitute an independent prognostic factor for OS (HR = 1.29, 95% CI 0.80-2.07, *P = 0.30*; Table S2). When cases with zero read counts for *GFRAL* were excluded from the analysis (n = 355), *GFRAL* positivity showed a borderline significant correlation with reduced OS (not reached *vs.* 801 days, *P = 0.058*; Figure S1C; HR = 3.09, 95% CI 0.91-10.52, *P = 0.07*; Table S1) and with poor prognosis of GC patients (HR = 3.25, 95% CI 0.95-11.17, *P = 0.06*; Table S2). In addition, the TCGA dataset showed that *RET* overexpression was associated with significantly shorter OS (2197 days *vs.* 661 days, *P < 0.0001*; Figure S1D), with HR calculation indicating an increase in relative risk of death from any cause of 1.99 (95% CI 1.39-2.83, *P = 0.0001*; Table S1). In the multivariate Cox analysis, *RET* overexpression remained an independent poor prognostic factor in terms of OS (HR = 1.92, 95% CI 1.33-2.75, *P = 0.0004*; Table S2). When we considered *RET* and *GDF15* together by Kaplan-Meier analysis, cases with both high *GDF15* expression and low *RET* expression had markedly longer OS than those with simultaneous low *GDF15* expression and high *RET* expression (2197 days *vs.* 588 days, *P = 0.0002*; Figure S1E). Furthermore, patients whose GC expressed both *RET* and *GDF15* at a high level had a visibly shorter OS compared to those whose GC expressed both *RET* and *GDF15* at a low level (*P = 0.161*; Figure S1F), and the survival benefit of *GDF15* overexpression markedly, but not significantly (*P = 0.274*) decreased when *GDF15* high expression was accompanied by *RET* overexpression (from 1686 days to 1095 days). Moreover, patients whose GC simultaneously expressed *RET* and *GFRAL* at a high level had significantly shorter survival time compared to those patients whose tumor tissue expressed both these markers at a low level (675 days *vs.* 2197 days, *P = 0.009*; Figure S1G). Finally, high combined expression of all selected markers: *GDF15+GFRAL+RET* significantly correlated with shorter

OS (675 days *vs.* 1811 days, *P = 0.006*; Figure S1H; HR = 1.56, 95% CI 1.13-2.15, *P = 0.01*; Table S1), and when adjusted for covariates, including pN and pT, it was an independent prognostic factor for OS (HR = 1.42, 95% CI 1.03-1.98, *P = 0.04*; Table S2).

GDF15, GFRAL and RET expression in gastric cancer: association with clinicopathological parameters

In TCGA cohort, the high expression level of *GDF15* was more often noted in G1-G2 (UQ: 41.51%; DESeq: 42.77%) gastric tumors than in G3 ones (UQ: 31.97%; DESeq: 27.87%), and the observed differences were marginally statistically significant or statistically significant, respectively for UQ (*P = 0.056*; Table S3) and DESeq (*P = 0.0025*; Table S4) normalized data. Moreover, the prevalence of positive *RET* was higher in gastric tumors classified as T3-T4 in comparison to those classified as T1-T2. This trend was found in both TCGA datasets, however the differences did not reach statistical significance (UQ: *P = 0.10*, Table S3; DESeq: *P = 0.15*, Table S4). The expression status of *GDF15*, *GFRAL* and *RET* was not associated with any remaining clinicopathological features (*P > 0.05*; Table S3 and S4).

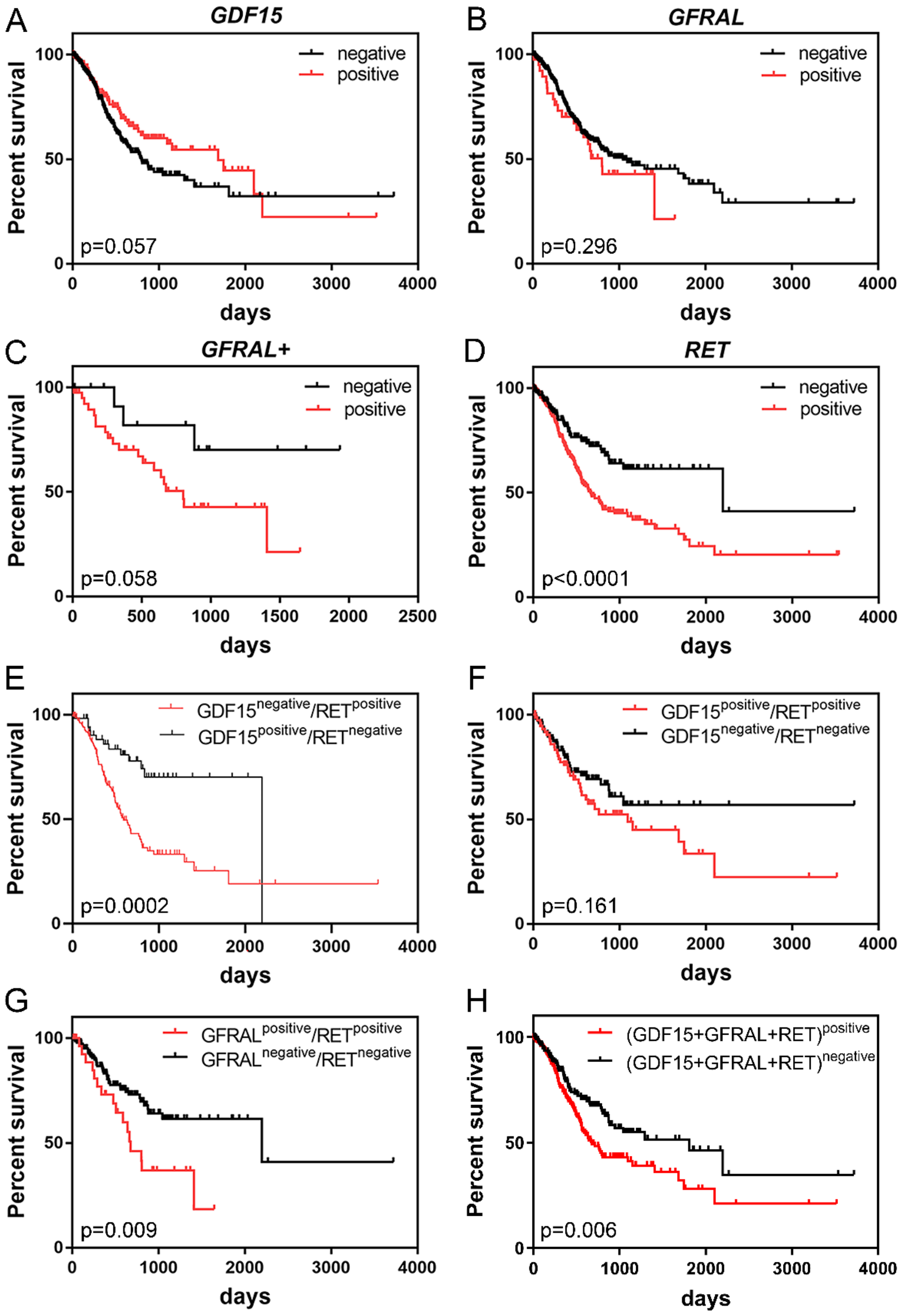

Figure S1. Kaplan-Meier curves displaying the survival time of GC patients depending on expression levels of GDF15 (A), GFRAL with (B) and GFRAL without cases with zero read counts (C), RET (D), the combination of GDF15 with RET (E, F, G) and the sum of GDF15, GFRAL and RET expression (H) prepared based on the DESeq-normalized RNA-seq data.

Table S1. Univariate Cox proportional hazards analysis for OS of TCGA patients with GC.

**Variable**	**Univariate analysis**			
	**HR**	**95% CI**		***P***
		**lower**	**upper**	
GDF15	0.72	0.51	1.01	0.06
GFRAL	1.29	0.80	2.06	0.30
GFRAL+	3.09	0.91	10.52	0.07
RET	1.99	1.39	2.83	**0.0001**
GDF15+GFRAL+RET	1.56	1.13	2.15	**0.01**
grading	1.44	1.03	2.02	**0.03**
pN status	2.09	1.39	3.14	**0.0004**
pT status	1.83	1.17	2.86	**0.01**
pM status	2.28	1.31	3.96	**0.003**

CI: confidence interval; GC: gastric cancer; HR: hazard ratio; OS: overall survival; TCGA: the Cancer Genome Atlas.
'GFRAL+' – cases with excluded zero read counts for *GFRAL*.
Significant *p*-values ($P < 0.05$) are indicated in bold.

Table S2. Multivariate Cox proportional hazards models for OS of TCGA patients with GC.

**Variable**	**Multivariate analysis: *GFRAL***				**Multivariate analysis: *GFRAL+***				**Multivariate analysis: *RET***				**Multivariate analysis: *GDF15+GFRAL+RET***			
	HR	95% CI lower	95% CI upper	*P*	HR	95% CI lower	95% CI upper	*P*	HR	95% CI lower	95% CI upper	*P*	HR	95% CI lower	95% CI upper	*P*
GFRAL	1.29	0.80	2.07	0.30	-	-	-	-	-	-	-	-	-	-	-	-
GFRAL+	-	-	-	-	3.25	0.95	11.17	0.06	-	-	-	-	-	-	-	-
RET	-	-	-	-	-	-	-	-	1.92	1.33	2.75	**0.0004**	-	-	-	-
GDF15+GFRAL+RET	-	-	-	-	-	-	-	-	-	-	-	-	1.42	1.03	1.98	**0.04**
pN status	1.84	1.19	2.82	**0.01**	0.77	0.29	2.02	0.59	1.78	1.17	2.72	**0.01**	1.80	1.18	2.76	**0.01**
pT status	1.45	0.90	2.34	0.13	0.81	0.28	2.39	0.71	1.44	0.90	2.32	0.13	1.45	0.90	2.33	0.12

CI: confidence interval; GC: gastric cancer; HR: hazard ratio; OS: overall survival; TCGA: the Cancer Genome Atlas.
p-values adjusted for pN, pT, and each marker separately or the sum of respective expression values of each marker (according to column captions); the sum was dichotomized < 18.11 or ≥ 18.11 using the *Evaluate Cutpoints* software.
'-' indicates variable was not included in multivariate analysis.
GFRAL+ – cases with excluded zero read counts for *GFRAL*.
Significant *p*-values ($P < 0.05$) are indicated in bold.

Table S3. Association of *GDF15*, *GFRAL*, *RET* expression and clinicopathological features in TCGA cohort of GC patients (UQ normalized data).

**Variable**	**n (%)**	***GDF15* expression**		**P value**	***GFRAL* expression**		**P value**	***RET* expression**		**P value**
	**n = 413**	negative n = 264	positive n = 149		negative n = 396	positive n = 17		negative n = 171	positive n = 242	
Grading										
G1-G2	159 (39.45)	93 (58.49)	66 (41.51)	0.056	155 (97.48)	4 (2.52)	0.21	73 (45.91)	86 (54.09)	0.18
G3	166 (60.55)	166 (68.03)	78 (31.97)		231 (94.67)	13 (5.33)		95 (38.93)	149 (61.07)	
pT status										
T1-T2	95 (23.69)	60 (63.16)	35 (36.84)	0.90	90 (94.74)	5 (5.26)	0.56	47 (49.47)	48 (50.53)	0.10
T3-T4	306 (76.31)	197 (64.38)	109 (35.62)		294 (96.08)	12 (3.92)		120 (39.22)	186 (60.78)	
pN status										
N0	125 (31.81)	79 (63.20)	46 (36.80)	>0.99	117 (93.60)	8 (6.40)	0.19	58 (46.40)	67 (53.60)	0.19
N1-N3	268 (68.19)	170 (63.43)	98 (36.57)		259 (96.64)	9 (3.36)		104 (38.81)	164 (61.19)	
pM status										
M0	370 (93.43)	237 (64.05)	133 (35.95)	0.30	355 (95.95)	15 (4.05)	>0.99	155 (41.89)	215 (58.11)	0.54
M1	26 (6.57)	14 (53.85)	12 (46.15)		25 (96.15)	1 (3.85)		9 (34.62)	17 (65.38)	

GC: gastric cancer; TCGA: the Cancer Genome Atlas; UQ normalization: upper quartile normalization.

Table S4. Association of *GDF15*, *GFRAL*, *RET* expression and clinicopathological features in TCGA cohort of GC patients (DESeq2 normalized data).

**Variable**	**n (%) n = 413**	***GDF15* expression**		**P value**	***GFRAL* expression**		**P value**	***RET* expression**		**P value**
		negative n = 272	positive n = 141		negative n = 370	positive n = 43		negative n = 167	positive n = 246	
Grading										
G1-G2	159 (39.45)	91 (57.23)	68 (42.77)	**0.0025**	144 (90.57)	15 (9.43)	0.74	69 (43.40)	90 (56.60)	0.41
G3	244 (60.55)	176 (72.13)	68 (27.87)		217 (88.93)	27 (11.07)		95 (38.93)	149 (61.07)	
pT status										
T1-T2	95 (23.69)	61 (64.21)	34 (35.79)	0.62	85 (89.47)	10 (10.53)	>0.99	45 (47.37)	50 (53.63)	0.15
T3-T4	306 (76.31)	205 (66.99)	101 (33.01)		273 (89.22)	33 (10.78)		118 (38.56)	188 (61.44)	
pN status										
N0	125 (31.81)	79 (63.20)	46 (36.80)	0.57	110 (88.00)	15 (12.00)	0.73	57 (45.60)	68 (54.40)	0.19
N1-N3	268 (68.19)	178 (66.42)	90 (33.58)		240 (89.55)	28 (10.45)		102 (38.06)	166 (61.94)	
pM status										
M0	370 (93.43)	246 (66.49)	124 (33.51)	0.20	332 (89.73)	38 (10.27)	0.74	150 (40.54)	220 (59.46)	>0.99
M1	26 (6.57)	14 (53.85)	12 (46.15)		23 (88.46)	3 (11.54)		10 (38.46)	16 (61.54)	

GC: gastric cancer; TCGA: the Cancer Genome Atlas.
Significant *p*-values ($P < 0.05$) are indicated in bold.
